# Late‐life hemoglobin A1C is associated with postmortem neuropathology in older adults with and without diabetes

**DOI:** 10.1002/alz.090116

**Published:** 2025-01-03

**Authors:** Roshni Biswas, Ana W. Capuano, Rupal I. Mehta, David A. Bennett, Zoe Arvanitakis

**Affiliations:** ^1^ Rush Alzheimer’s Disease Center, Rush University Medical Center, Chicago, IL USA; ^2^ Rush University Medical Center, Chicago, IL USA; ^3^ Rush Alzheimer’s Disease Center, Chicago, IL USA; ^4^ Department of Neurological Sciences, Rush University Medical Center, Chicago, IL USA

## Abstract

**Background:**

Hemoglobin A1C (A1C) is a measure of long‐term glycemic control. In a previous study using a single measure of A1C, we showed that it is related to postmortem cerebrovascular pathology. Here, we use annually collected A1C data to study the relationship of A1C average and variability over time with neuropathology in a large number of older adults with and without diabetes.

**Method:**

Our study included annually collected longitudinal data on 820 older women and men (mean years of follow‐up 4.0 years, SD = 4.5) who had at least two measures of A1C and two neuropsychological evaluations, and who agreed to autopsy. We excluded those with dementia at the time of their first A1C measurements. Systematic postmortem neuropathologic evaluations documented the presence and size (macroscopic and microscopic) of brain infarcts. Modified silver stain‐based Alzheimer’s disease (AD) measures included global scores. We evaluated the association of A1C mean and variability (measured as the coefficient of variation of A1C measurements) during the study, with neuropathology, using a series of models (e.g., linear models, logistic regression, and extensions) adjusting for age at death, sex, and education.

**Result:**

Participants (mean age‐at‐death = 90.9 [SD = 6.5] years; education = 15.8 [SD = 3.3] years;75% women) had an average of 5.7 (SD = 2.8) annually collected A1C measurements. The average person‐specific mean and coefficient of variation of A1C during the study were 6.0 (SD = 0.64) and 0.04 (SD = 0.03), respectively. Analyses showed that the mean A1C was associated with an increased odds of macroinfarcts (OR = 1.27; 95%CI:1.01‐1.59), but not microinfarcts. Notably, mean A1C was inversely associated with global AD pathology (β = ‐0.08, p‐value = 0.01). Variability in A1C was not associated with neuropathology.

**Conclusion:**

Using longitudinal A1C measures, we confirmed that A1C level is associated with cerebrovascular pathology and identified an inverse association with AD pathology. However, A1C variability over time showed no associations. Further studies are needed to understand the relationship of A1C to neuropathologies.

